# Essential oils against bacterial isolates from cystic fibrosis patients by means of antimicrobial and unsupervised machine learning approaches

**DOI:** 10.1038/s41598-020-59553-8

**Published:** 2020-02-14

**Authors:** Rino Ragno, Rosanna Papa, Alexandros Patsilinakos, Gianluca Vrenna, Stefania Garzoli, Vanessa Tuccio, ErsiliaVita Fiscarelli, Laura Selan, Marco Artini

**Affiliations:** 1 Rome Center for Molecular Design, Department of Drug Chemistry and Technology, Sapienza University, p.le Aldo Moro 5, 00185 Rome, Italy; 2 Alchemical Dynamics s.r.l, 00125 Rome, Italy; 3 Department of Public Health and Infectious Diseases, Sapienza University, p.le Aldo Moro 5, 00185 Rome, Italy; 4 Department of Drug Chemistry and Technology, Sapienza University, p.le Aldo Moro 5, 00185 Rome, Italy; 5 Laboratories and Pediatrics Departments, Children’s Hospital and Institute Research Bambino Gesù, Rome, 00165 Italy

**Keywords:** Antimicrobial resistance, Clinical microbiology

## Abstract

Recurrent and chronic respiratory tract infections in cystic fibrosis (CF) patients result in progressive lung damage and represent the primary cause of morbidity and mortality. *Staphylococcus aureus* (*S. aureus*) is one of the earliest bacteria in CF infants and children. Starting from early adolescence, patients become chronically infected with Gram-negative non-fermenting bacteria, and *Pseudomonas aeruginosa* (*P. aeruginosa*) is the most relevant and recurring. Intensive use of antimicrobial drugs to fight lung infections inevitably leads to the onset of antibiotic resistant bacterial strains. New antimicrobial compounds should be identified to overcome antibiotic resistance in these patients. Recently interesting data were reported in literature on the use of natural derived compounds that inhibited *in vitro S. aureus* and *P. aeruginosa* bacterial growth. Essential oils, among these, seemed to be the most promising. In this work is reported an extensive study on 61 essential oils (EOs) against a panel of 40 clinical strains isolated from CF patients. To reduce the *in vitro* procedure and render the investigation as convergent as possible, machine learning clusterization algorithms were firstly applied to pick-up a fewer number of representative strains among the panel of 40. This approach allowed us to easily identify three EOs able to strongly inhibit bacterial growth of all bacterial strains. Interestingly, the EOs antibacterial activity is completely unrelated to the antibiotic resistance profile of each strain. Taking into account the results obtained, a clinical use of EOs could be suggested.

## Introduction

Cystic fibrosis (CF), one of the most common lethal genetic disorders in Caucasian population, is inherited as an autosomal recessive disease and affects 70.000 persons worldwide (Cystic Fibrosis Foundation, CFF). The defective gene, identified in 1989, is the Cystic Fibrosis Transmembrane Conductance Regulator (CFTR) that is carried by 4% of persons (among Caucasians). Since CFTR encodes for a chloride channel of the epithelial cell surface, CF patients manifest a variety of multi-organ problems due to the alteration of sodium and chloride secretion across cell membranes and the subsequent luminal dehydration^1^. The impairment of mucociliary clearance, which should remove all microbes entering the airways, leads to the production of a thick and dehydrated mucus in the CF lung, which promotes the airway chronic bacterial colonization^2^.

The microbiology of CF respiratory tract is peculiar. In the early stage of life, it is characterized by the prevalence of the Gram-positive bacterium *Staphylococcus aureus* (*S. aureus*). Overall, in 2017 more than half of affected individuals had at least one culture positive for methicillin sensitive *S. aureus* (MSSA). The highest prevalence of methicillin resistant *S. aureus* (MRSA) occurs in individuals between the ages of 10 and 30, while MSSA reaches the peak among patients younger than 10 (Cystic Fibrosis Foundation. 2017. Patient Registry Annual Data Report. https://www.cff.org/Research/Researcher-Resources/Patient-Registry/2017-Patient-Registry-Annual-Data-Report.pdf).

In early adolescence, CF patients’ lung becomes chronically infected with Gram-negative non-fermenting bacteria. Among these, *Pseudomonas aeruginosa* (*P. aeruginosa*) is the most relevant and recurring, so that 30% of CF children and up to 80% of CF adults (25 years old and older) have lungs chronically colonized by this pathogen^3^. *P. aeruginosa* isolated from respiratory secretions demonstrates great phenotypic diversity and develops genetic mutations over time to adapt and survive in the complex environment of the CF airway^4^. *P. aeruginosa* mucoid phenotype, defined by the exopolysaccharide alginate overproduction within lungs of CF patients, is a hallmark of chronic infection and predictive of poor prognosis. Indeed, mucoid *P. aeruginosa* has also been associated with failure of eradication and, compared to non-mucoid counterpart, exhibits enhanced resistance to multiple antibiotics and host immune effectors^5^.

Due to current therapeutic treatments, life expectancy for CF patients has consistently grown, reaching a median life of 40 years. Assuming a positive trend of clinical care improvements at the actual rate, CF patients born in 2010 are expected to live up to 50 years of age^6^.

The intensive use of antimicrobial drugs to fight lung infections inevitably leads to the onset of antibiotic resistant bacterial strains. New antimicrobial compounds should be identified to overcome antibiotic resistance during the treatment of CF lung infections.

Recent investigation has disclosed a few small molecules, such as peptides or mannosides, showing promising efficacy in prevention and treatment of both bacterial and fungal biofilm infection *in vivo*^7^. Nevertheless, due to their mechanism of action based on a specific binding to a main target, the use of small molecules is known to select more and more resistant strains^8^. Interestingly in the recent literature appeared some reports on the use of natural derived compounds that showed *in vitro* the potentiality to inhibit the development of CF associated infections^9–12^. In particular essential oils seemed to be the most promising agents among tested natural compounds^10,11^. In this study is reported an extensive study on 61 essential oils (EOs) against a panel of 40 bacterial strains isolated from CF patients (see Table 1).

**Table 1:** Classification of bacterial strains based on their biofilm formation ability.

Bacterial strains	Biofilm producer	Bacterial strains	Biofilm producer
6538P*	STRONG	PAO1*	STRONG
25923*	STRONG	PA14*	STRONG
1S	WEAK	21P	STRONG
2S	MODERATE	22P	NP
3S	WEAK	23P	MODERATE
4S	WEAK	24P	NP
5S	WEAK	25P	WEAK
6S	WEAK	26P	NP
7S	MODERATE	27P	NP
8S	WEAK	28P	NP
9S	WEAK	29P	NP
10S	MODERATE	30P	WEAK
11S	WEAK	31P	MODERATE
12S	WEAK	32P	WEAK
13S	WEAK	33P	NP
14S	WEAK	34P	WEAK
15S	MODERATE	35P	WEAK
16S	WEAK	36P	WEAK
17S	MODERATE	37P	STRONG
18S	WEAK	38P	MODERATE
19S	WEAK	39P	WEAK
20S	MODERATE	40P	WEAK

For *S. aureus*, results were analysed according to Cafiso *et al*.^14^; for *P. aeruginosa* classification was based on Perez *et al*.^15^.

NP: non biofilm producer.

*Reference strains.

To reduce the *in vitro* procedure and to render the investigation as convergent as possible the following workflow was followed. Unsupervised machine learning algorithms and techniques, as implemented in python language^13^, were firstly applied to pick-up a fewer number of representative strains (RS) among the panel of 40. To this aim, a number of categorical descriptors were collected and used to cluster the CF isolated strains. The clusters’ centroids indicated the RS to be investigated for their susceptibility to a list of commercial EOs at fixed doses. Three EOs showed a great efficacy to reduce the microrganisms growth and were therefore promptly assayed against all the available clinical isolates. The three EOs confirmed the initial assumption demonstrating their ability to inhibit bacterial growth. Gas chromatography coupled with mass spectrometry (GC/MS) was then performed on the three EOs to investigate on the likely chemical components mainly responsible for the antibacterial activity.

## Results

### Characterization of biofilm formation of clinical bacterial strains

**Figure 1:**
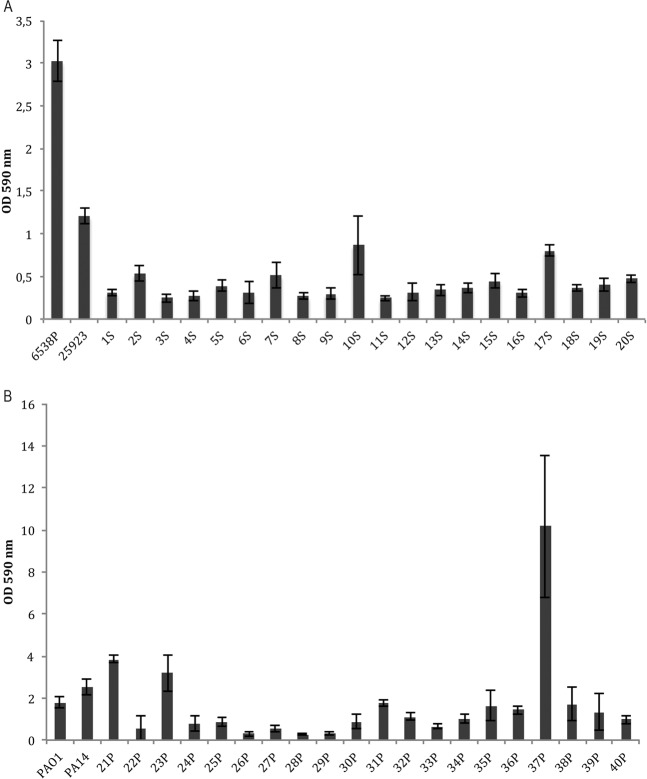
Biofilm formation of *S. aureus* clinical and reference strains (**A**) and *P. aeruginosa* clinical and reference strains (**B**). The biofilm formation was evaluated after 18 h incubation in polystyrene plates at 37 °C. The data are reported as OD 590 nm after crystal violet staining. Each data point represents the mean ± SD of four independent samples.

Clinical bacterial strains were investigated for their ability to produce biofilm (Fig. 1). Biofilm formation was evaluated at 37 °C in BHI for 18 h as described in Material and Methods section.

Biofilm formation was also evaluated for four reference strains included in the experimental plan. Figure 1A reports biofilm formation of bacterial strains belonging to *S. aureus* species. Clinical strains, named from 1S to 20S, were classified as “*weak*” or “*moderate*” biofilm producers according to Cafiso and coworker, 2007^14^. Both reference strains for *S. aureus* species are strong biofilm producers. Figure 1B reports biofilm formation of bacterial strains belonging to *P. aeruginosa* species. Clinical strains, named from 21P to 40P, were classified as: “*non producer*”, “*weak*”, “*moderate*” and “*strong*” biofilm producers according to Perez and Barth, 2011^15^ (Table 1).

### Selection of representative microorganisms by machine learning

The 40 selected strains were divided accordingly to the main strains families into *S. aureus* and *P. aeruginosa* dataset and imported into a python pandas dataframe. The principal components analysis (PCA) indicated that 90% of the variance is explained by the first 10^th^ principal components (PCs) (Fig. 1S). Nevertheless graphical inspection of the PC1 versus PC2 scores and loadings plots indicated the PCs as potential new variables to cluster the datasets (Fig. 2S). As a matter of fact, application of the Silhouette Analysis^16^ coupled with the *k*-means clustering^17^ to the first 2 PCs indicated the optimal number of clusters to be 6 and 3 for the *P. aeruginosa* and *S. aureus* strains, respectively (Figs. 3S and 4S). For each cluster, the nearest datapoint to cluster centroid was selected yielding to a selection of representative strains to be screened with the commercial EOs. Analysis of data revealed the six samples, precisely 22P, 25P, 26P, 27P, 37P and 39P as the representatives for *P. aeruginosa*, whereas samples 4S, 5S and 19S were selected for *S. aureus*.

### Antimicrobial activity of EOs on *P. aeruginosa* and *S. aureus* clinical strains from cystic fibrosis patients

Essential oils were tested for their ability to inhibit bacterial growth of *P. aeruginosa* and *S. aureus* clinical and reference strains. Analysis was performed on three representative *S. aureus* strains and six representative *P. aeruginosa* strains, previously selected by machine learning analysis. EOs were tested at a concentration of 1% v/v (Table 2). Several EOs have shown antimicrobial activity on many bacterial strains. It is worthy to note that the *P. aeruginosa* reference strain PAO1 is the most resistant to the action of EOs, since it was inhibited by only four EOs.

**Table 2:** Antimicrobial activity of EOs listed in Table 2S on representative clinical strains and reference strains of *S. aureus* and *P. aeruginosa*.

Eos ID	6538P	25923	4S	5S	19S	PaO1	PA14	22P	25P	26P	27P	37P	39P
1	1%	1%	1%										
2													
3								1%				1%	
4	1%	1%	1%	1%	1%			1%				1%	
5													
6		1%	1%	1%	1%								
7													
8													
9	1%	1%	1%	1%	1%		1%	1%	1%	1%		1%	1%
10													
11													
12													
13													
14	1%	1%	1%	1%	1%	1%	1%		1%		1%	1%	1%
15													
16													
17													
18													
19													
20													
21													
22	1%	1%	1%	1%	1%	1%	1%	1%	1%	1%	1%	1%	1%
23													
24													
25													
26													
27													
28													
29													
30													
31													
32	1%	1%	1%	1%	1%	1%	1%	1%	1%	1%	1%	1%	1%
33													
34													
35													
	*follows*												
36													
37	1%	1%	1%	1%	1%			1%				1%	1%
38												1%	
39	1%	1%	1%	1%	1%	1%	1%	1%	1%	1%	1%	1%	1%
40													
41													
42													
43													
44													
45													
46	1%	1%	1%	1%	1%							1%	1%
47													
48	1%	1%	1%	1%	1%		1%						1%
49													
50													
51													
52				1%	1%								
53													
54	1%				1%								
55													
56													
57													
58													
59	1%	1%	1%	1%	1%							1%	1%
60				1%	1%							1%	1%
61	1%			1%									1%

This analysis allowed to identify three EOs active against all the representative strains used, namely cade essential oil (22 in Table 2S, CEO), birch essential oil (32 in Table 2S, BEO) and Ceylon cinnamon peel essential oil (39 in Table 2S, CCPEO). Thus, these 22, 32 and 39 EOs were tested on all clinical bacterial strains. Results summarized in Table 3 confirmed that BEO, CEO and CCPEO exerted a strong and effective bactericidal potency on all tested clinical strains.

**Table 3:** Antimicrobial activity corresponding to minimal bactericidal concentration of previously selected EOs on all 40 clinical isolates.

Bacterial strains	CEO	BEO	CCPEO	Bacterial strains	CEO	BEO	CCPEO
ATCC6538P	1%	1%	1%	PA O1	1%	1%	1%
ATCC25923	1%	1%	1%	PA 14	1%	1%	1%
SA01	1%	1%	1%	PA21	1%	1%	1%
SA02	1%	1%	1%	PA22	1%	1%	1%
SA03	1%	1%	1%	PA23	1%	1%	1%
SA04	1%	1%	1%	PA24	1%	1%	1%
SA05	1%	1%	0.1%	PA25	1%	1%	1%
SA06	1%	1%	1%	PA26	1%	1%	1%
SA07	1%	1%	1%	PA27	1%	1%	1%
SA08	1%	1%	1%	PA28	1%	1%	1%
SA09	1%	1%	1%	PA29	1%	1%	1%
SA10	1%	1%	1%	PA30	1%	1%	1%
SA11	1%	1%	1%	PA31	1%	1%	1%
SA12	1%	1%	1%	PA32	1%	1%	1%
SA13	1%	1%	1%	PA33	1%	1%	1%
SA14	1%	1%	1%	PA34	1%	1%	1%
SA15	1%	1%	1%	PA35	1%	1%	1%
SA16	1%	1%	1%	PA36	1%	1%	1%
SA17	1%	1%	1%	PA37	1%	1%	1%
SA18	1%	1%	1%	PA38	1%	1%	1%
SA19	1%	1%	1%	PA39	1%	1%	1%
SA20	1%	1%	1%	PA40	1%	1%	1%

### Chemical composition analysis of active selected essential oils

The results of GC and GC-MS analyses of the essential oils are reported in Table 3S–5S. In the BEO, 21 components were identified and the major constituents were δ-cadinene, calamenene and creosol (22.2%, 15.2% and 12.8% respectively) (Table 3S). The chemical composition of CCPEO was characterized by the presence of 19 compounds and by a high amount of cinnamaldehyde (49.4%) followed by eugenol (21.2%) (Table 4S). The chemical composition of the CEO indicated 21 components and the most abundant were delta-cadinene (27.7%), calamenene (14.8%) and creosol (12.6%) (Table 5S). At first glance the chemical composition of the CEO seems very similar to that of BEO as the main compounds showed comparable percentages. Among the minor components of CEO, α-selinene (2.2%), aromadendrene (1.1%) and gleenol (1.1%) were found, whereas isoledene (5.7%) was found in BEO. At a deeper analysis the chemical qualitative profiles were compared and a 0.62 tanimoto index was calculated, thus indicating that although displaying a similar chromatogram the two EOs are indeed different. EOs producer was also inquired and their technical staff confirmed the two oils sharing high similarity quantitative profile in the main constituents.

## Discussion

Long-term administration of antibiotics to prevent and treat airway infections in CF patients has been shown to be associated with the emergence of multi-drug (MDR) antimicrobial resistant microorganisms^18^.

In particular, *mecA/mecC* genes acquisition in *S. aureus* and accumulation of resistance mechanisms after antibiotic exposure in *P. aeruginosa*, both key pathogens in CF lung, are a concern in this context^19,20^.

Multidrug resistance significantly limits effective therapeutic options, affecting clinical outcome and prognosis of patients. For this reason, the identification and development of new antibacterial agents is fundamental to improve survival and quality of life of individuals with CF. Therefore the development of antimicrobial agents provided with novel molecular mechanisms that may allow to control bacterial infectious diseases without diffusing antibacterial resistance is desirable^21^.

Unsupervised Machine Learning algorithms^13^ applied to a panel of 40 strains of *S. aureus* and *P. aeruginosa* isolated from CF patients, led to select fewer representative strains using phenotypical and genotypical characteristics as categorical descriptors. Therefore, the antibacterial activity of all tested EOs was initially assessed on 9 selected bacterial strains: six representative strains for *P. aeruginosa* and 3 representative strains for *S. aureus*. The activity of all 61 EOs was also assessed on reference strains. Antimicrobial assays led to identify 3 EOs (CEO, BEO and CCPEO) out of the tested 61, that exhibited the highest antibacterial activity on the previously selected bacterial strains and reference ones. The antibacterial activity of the 3 selected EOs was then extended to all strains of both species. Interestingly all three EOs showed an utmost antimicrobial potency on all studied strains. Nothing can be yet ruled out on the chemical compounds’ role. Future studies involving machine learning application^10,11^, will be devoted to investigate the importance of chemical constituent either on biofilm modulation or in antibacterial potencies.

Several papers aimed at elucidating the antimicrobial mechanism of action of EOs. For example, cinnamaldehyde, the major component of cinnamon, is able to disrupt the transmembrane potential of *P. aeruginosa*^22^.

Furthermore, EOs of different origin (lavender, lemongrass, marjoram, peppermint, tea tree and rosewood) show antimicrobial activity against *Burkholderia cepacia complex* by inducing changes in membrane fatty acid composition, followed by membrane disruption^23^. Also, EO from Alluaudia procera was active against *S. aureus* ATCC25923, a multi-resistant strain^24^. Reported data confirmed the possibility to use EOs as therapeutic strategies in multi-resistant strains probably due to the heterogeneous composition of the oils themselves.

Notably, in this work we found EOs antibacterial activity unrelated to the antibiotic resistance profile of each strain.

This observation is of particular relevance as it suggests the EOs potential uses by topical administration without taking into account the complexity of drug resistance profile of the microbiota in each single patient.

In conclusion the approach herein applied allowed to minimize the experimental steps and it was possible to identify the most promising EOs on the basis of probabilistic evaluations that confirmed their wide spectra of antibacterial potency with a reduced set of experiments.

From a literature survey (www.scopus.com, accessed 2019 December 13, keywords: essential oil, antibacterial activity and resistance) no evidence of resistance to EOs antibacterial activity has yet been reported. This is a characteristic particularly relevant for antibacterial candidates to be administered for a chronic disease such as CF.

Indeed some papers report an increase of susceptibility to antibiotics after treatment with essential oils^25,26^.

Although a pletora of publications did not show development of resistance to EOs, a very recent publication suggested the induction of efflux pumps and multidrug resistance in *P. aeruginosa* by Cinnamaldehyde, the main component of cinnamon^27^. Therefore, in light of the recent reports, much still needs to be clarified on the effect of essential oils on bacterial multi-drug resistance.

## Methods

### Ethics approval and informed consent

The approval for this research was granted by the Ethics Committee of Children’s Hospital and Institute Research Bambino Gesù in Rome, Italy (No 1437_OPBG_2017 of July 2017), and it was performed according to the principles of the Helsinki Declaration. Informed consent was obtained from all individual participants and all parents/legal guardians included in the study.

### Clinical isolates from CF patients

In this study were used 40 bacterial strains (20 *S. aureus*, 20 *P. aeruginosa*) obtained from respiratory specimens of 30 CF patients (13 males, 17 females; medium age 20.5) in follow-up at Pediatric Hospital Bambino Gesù (OPBG) of Rome, Italy. In particular, 27 bacterial strains were isolated from sputum, 11 from hypopharyngeal suction and 2 from throat swabs (Tables 4 and 5). As reference strains were used: *S. aureus* ATCC 6538P (6538P) and *S. aureus* ATCC 25923 (25923) commonly recognized as reference strains for antimicrobial testing; *P. aeruginosa* PAO1 (PAO1) and *P. aeruginosa* PA14 respectively recognized as moderately and highly virulent^28^.

**Table 4:** The 20 *Staphylococcus aureus* clinical isolates and their characterization by several properties.

ID pt	ID	SAM	Date	Str	Ph	QUIN	B	ER	CLI	LIN	RCLI	CF	CPA	GEN
1	1S	ESP	10/11/2006	MRSA	SCV	R	S	Nt	R	S	N	Cp		J
2	2S	ESP	11/22/2007	MRSA	SCV	R	S	Nt	R	S	N	Ca	X	N
3	3S	ESP	1/15/2009	MRSA	SCV	S	S	Nt	S	S	N		X	E
4	4S	AT	2/20/2009	MRSA	—	S	S	Nt	S	S	P			A
5	5S	ESP	11/13/2009	MRSA	—	R	S	Nt	R	S	N	Sp		C
6	6S	AT	1/10/2011	MRSA	—	R	S	Nt	R	S	P			K
7	7S	ESP	4/4/2011	MRSA	—	R	S	Nt	R	S	N	Ca	X	D
8	8S	AT	7/22/2013	MRSA	—	R	S	Nt	S	S	N			I
9	9S	ESP	1/15/2014	MRSA	—	S	S	Nt	R	S	P	Ca	X	C
10	10S	AT	1/29/2015	MRSA	—	S	S	Nt	R	S	N	Ca/Cd/Pb		G
11	11S	AT	6/15/2017	MSSA	—	S	S	R	R	S	P			C
12	12S	AT	6/15/2017	MSSA	—	S	S	R	R	S	P			U
13	13S	AT	5/23/2017	MSSA	—	I	S	I	I	S	N	Sa		B
14	14S	AT	5/25/2017	MSSA	—	S	S	S	S	S	N			C
15	15S	AT	5/24/2017	MSSA	—	S	S	R	S	S	N		X	C
16	16S	AT	5/26/2017	MSSA	—	S	S	R	R	S	N			H
17	17S	AT	5/25/2017	MSSA	—	S	S	R	R	S	N	Af		M
18	18S	ESP	5/24/2017	MSSA	—	S	S	R	R	S	P	Ca	X	C
19	19S	ESP	6/15/2017	MSSA	—	S	S	R	R	S	P		X	L
20	20S	ESP	5/19/2017	MSSA	—	R	S	R	R	S	P		X	F

ID pt: patient identification; ID: strain code; SAM:Sample; Date: Date of collection; Str:Strain; Ph: phenotype; QUIN: quinolones; B: Trimethoprim/Sulfamethoxazole; ER: Erythromycin; CLI: Clindamycin; LIN: linezolid; RCLI: Inducible Clindamycin resistance; CF: Fungal Co-infection; CPA: *P.* *aeruginosa* co-infection; GEN: pts genotype; Esp: sputum; AT: hypopharyngeal suction; MRSA: Methicillin Resistant *S*. *aureus*; MSSA: Methicillin Sensitive *S*. *aureus*; SCV: Small colony variant; R: Resistant;S: Susceptible; I: Intermediate; N: Negative; Nt: non-tested; Af: *Aspergillus fumigatus*; Ca: *Candida albicans*; Cp: *Candida parapsilosis*; Sp: *Scedosporium prolificans*; Cd: *Candida dubliniensis*; Pb: Pseudoallescheria boydii; Sa: *Scedosporium apiospermum*. X: denotes positive for this feature; -: denotes common phenotype. See Table 6 showing the correlation between letter code, CFTR gene mutation of the patient and bacterial strain isolated from the same patient.

**Table 5:** The 20 *Pseudomonas aeruginosa* clinical isolates and their characterization by several properties.

ID pt	ID	SAM	date	Str	Ph	CAR	PTC	AM	QUIN	MB	CEF	COL	1St	E	L	CF	CSA	GEN
21	21P	ESP	8/8/2006	PA MDR	s	R	R	R	R	R	R	S			X			E
21	22P	ESP	1/11/2017	PA MDR	w	R	S	R	R	S	R	S			X			E
22	23P	ESP	6/24/2005	PA MDR MBL+	sm	R	S	R	R	S	R	S		X				B
22	24P	ESP	3/27/2017	PA MDR MBL+	s	R	S	R	R	S	R	S			X	Ca/Cl		B
23	25P	AT	9/3/2010	PA	sm	S	S	I	S	I	S	S	X					B
24	26P	TF	8/27/2008	PA	i	S	S	S	S	I	S	S	X					G
24	27P	AT	1/31/2017	PA	sm	S	S	S	S	S	S	S			X		X	G
25	28P	ESP	5/24/2012	PA	sm	S	S	S	S	S	S	S	X					U
25	29P	AT	9/13/2017	PA	m	S	S	S	S	S	S	S			X			U
9	30P	ESP	9/6/2010	PA	i	S	S	S	S	I	S	S	X					B
9	31P	ESP	1/11/2017	PA	m	S	S	S	R	S	S	S			X		X	B
26	32P	AT	12/5/2006	PA	sm	S	S	R	S	I	S	S	X					F
26	33P	AT	12/28/2016	PA	m	S	S	S	S	I	S	S			X	Ca	X	F
27	34P	ESP	5/11/2005	PA	i	MP I/IP R	S	S	CI S/LE R	I	S	S	X			Ca		D
27	35P	ESP	3/29/2017	PA MDR	sm	R	R	R	R	I	R	S			X			D
28	36P	TF	2/11/2008	PA	sm	S	S	S	S	I	S	S		X				A
28	37P	AT	2/22/2017	PA	m	MP S/IP R	S	R	S	S	R	S			X			A
29	38P	ESP	3/7/2006	PA MDR	s	R	R	R	R	R	R	S			X			B
29	39P	ESP	1/25/2017	PA MDR	m	R	R	R	R	I	R	S			X			B
30	40P	AT	7/1/2013	PA	i	S	S	S	S	S	S	S	X			Ca	X	C

ID pt: patient identification;ID: strain code;SAM: Sample; Date: Date of collection; Str: Strain; Ph: Phenotype; CAR: Carbapenems; MP: Meropenem; IP: Imipenem; PTC: Piperacillin/tazobactam; AM:

Aminoglycosides; QUIN: Quinolones; CI: Ciprofloxacin; LE: Levofloxacin; MB: Monobactam; CEF: Cephalosporins; COL: Colistin; 1 St: *P. aeruginosa* first isolate; E: *P. aeruginosa* early isolate; L: *P. aeruginosa* late isolate; CF: Fungal co-infection; CSA: *S. aureus* co-infection; Gen: pts genotype; BP: Biofilm Producer; Esp:sputum; AT: hypopharyngeal suction; TF: throat swabs; PA: *P. aeruginosa*; PA MDR: *P. aeruginosa* multi-drug resistant; PA MBL+: *P. aeruginosa* Metallo-Beta-Lactamases producing; s: small colony phenotype; w- wrinkled colony surface; m: mucoid colony; i: irregular colony edges; sm: smooth phenotype; R: Resistant; S: Susceptible; I: Intermediate; CA: *Candida albicans;* CL: *Candida lusitaniae;* X: denotes positive for the feature. See Table 6 showing the correlation between letter code, CFTR gene mutation of the patient and bacterial strain isolated from the same patient.

Patients were treated according to current standards of care^29^ with at least four microbiological controls per year. Informed consent was obtained from all subjects aged 18 years and older and from parents of all subjects under 18 years of age prior to enrolment.

Microbiological cultures have been performed according to approved Guidelines, using selective media, manual and automatic systems (API20NE, Vitek2, MALDI-TOF mass spectrometry) for isolates identification and 16S rRNA sequencing to assess ambiguous identifications.

The strains were selected from a local bacteria collection including about 10.000 CF bacterial isolates.

The species *S. aureus* and *P. aeruginosa* have been chosen for their clinical relevance in the natural history of CF disease, since they are related to a worst prognostic impact compared to other pathogens whose role is still under discussion.

In order to represent the complexity of CF lung microbiota population attending OPBG Center, a selection of specific strains with different phenotypic and biochemical features has been performed. The strains’ characteristics are described in Tables 4 and 5.

### Qualitative description of the clinical isolates

Twenty *S. aureus* strains with a different susceptibility profile, belonging to 20 CF patients, were selected: 10 Methicillin-Sensitive (MSSA) and 10 Methicillin-Resistant (MRSA). Among the MRSA strains, three *S. aureus* with phenotypic “small colony variants” (SCVs) have been chosen, characterized by slow growth of small, unpigmented, non-haemolytic colonies.

Antimicrobial susceptibility profiles of MSSA and MRSA isolates were defined by automatic system Vitek2 (Biomerieux, France) or manual system E-test (Liofilchem, Italy). In particular, susceptibility to quinolones (ciprofloxacin, levofloxacin), trimethoprim/sulfamethoxazole, erythromycin, clindamycin, linezolid was assessed, according to EUCAST (www. EUCAST.org) criteria. Moreover, the clindamycin-inducing resistance test (40% positive test) was performed to classify *S. aureus* isolates that could develop acquired resistance to erythromycin or other macrolides during therapy with this antibiotic (Table 4)^30^.

Twenty *P. aeruginosa* isolates belonging to 11 CF patients were also selected (Table 5). The selected strains had been categorized as first, early and late isolates. In particular, seven strains have been associated to first acquisition of *P. aeruginosa* (first strains), 2 strains have been isolated 1 year after the first acquisition (early strains) and 11 strains have been isolated at least 5 years after the onset of chronic colonization (late strains).

Moreover, different phenotypes (mucoid, wrinkle surface, irregular edges or smooth) and strains with different antibiotic susceptibility patterns, e.g. *P. aeruginosa* producing Metallo-Beta-Lactamases (MBL)^31^ or *P. aeruginosa* multi-drug resistant (MDR), have been selected.

Susceptibility testing to carbapenems (imipenem, meropenem), piperacillin/tazobactam, aminoglicosides (tobramycin, amikacin), quinolones (ciprofloxacin, levofloxacin), monobactam (aztreonam), and cephalosporins (ceftazidime, cefepime) was carried out by Minimum Inhibitory Concentration (MIC) determined by E-test on Mueller Hinton (MH) agar plates, according to EUCAST criteria. The colistin MIC values were evaluated by Broth Microdilution (ComASP Colistin Liofilchem, Italy); 35% of *P. aeruginosa* isolates were MDR (i.e. resistant to three or more classes of antimicrobials)^32^ (Table 3). Table 1 of Supplementary Materials reports the percentage of bacterial strains resulted sensitive or resistant to different classes of antibiotics here tested (Table 1S).

Co-infection by bacterial (*P. aeruginosa*/*S. aureus*) and fungal agents (*Aspergillus fumigatus*/*Candida albicans*/*Candida parapsilosis*/*Candida dubliniensis*/*Candida lusitaniae*/*Scedosporium prolificans*/*Scedosporium apiospermum*//*Pseudoallescheria boydii*) was also evaluated for each patient (Tables 4 and 5).

Table 6 reports letters’ code correspondence for the strains associated genotype reported in Tables 4 and 5.

**Table 6:** Table shows the correlation between letter code, CFTR gene mutation of the patient and bacterial strain isolated from the same patient.

Code	Genotype	ID strain
A	621+1G > T/R553X	4S
B	F508del/1717-1G- > A	13S
C	F508del/F508del	5S
C	F508del/F508del	9S
C	F508del/F508del	11S
C	F508del/F508del	14S
C	F508del/F508del	15S
C	F508del/F508del	18S
D	F508del/G1244E	7S
E	F508del/G542X	3S
F	F508del/L1077P	20S
G	F508del/R1162X	10S
H	F508del/R117L + L997F	16S
I	F508del/R585X	8S
J	F508del/W1282X	1S
K	G542X/3271 + 42A/T	6S
L	L636P/P499A	19S
M	N1303K/2184insA	17S
N	Q220X/A1006E	2S
U	None	12S
A	F508del/E193K	36P
A	F508del/E193K	37P
B	F508del/F508del	23P
B	F508del/F508del	24P
B	F508del/F508del	25P
B	F508del/F508del	30P
B	F508del/F508del	31P
B	F508del/F508del	38P
B	F508del/F508del	39P
C	F508del/G542X	40P
D	F508del/l1234V	34P
D	F508del/l1234V	35P
E	N1303K/3849 + 10kbC > T	21P
E	N1303K/3849 + 10kbC > T	22P
F	R347P/L571S	32P
F	R347P/L571S	33P
G	W1282X/2789 + 5G- > A	26P
G	W1282X/2789 + 5G- > A	27P
U	None	28P
U	None	29P

### Biofilm production assay

The quantification of biofilm production was based on microtiter plate biofilm assay (MTP) as reported in literature^12^. Briefly, the wells of a sterile 96-well flat-bottomed polystyrene plate were filled with 100 µL of the appropriate medium. 1/100 dilution of overnight bacterial cultures was added into each well (about 0.5 OD 600 nm). The plates were incubated aerobically for 18 h at 37 °C. Biofilm formation was measured using crystal violet staining. After incubation, planktonic cells were gently removed; each well was washed three times with double-distilled water and patted dry with a piece of paper towel in an inverted position. To quantify biofilm formation, each well was stained with 0.1% crystal violet and incubated for 15 min at room temperature, rinsed twice with double-distilled water, and thoroughly dried. The dye bound to adherent cells was solubilized with 20% (v/v) glacial acetic acid and 80% (v/v) ethanol. After 30 min of incubation at room temperature, OD590 was measured to quantify the total biomass of biofilm formed in each well. Each data point is composed of 4 independent experiments, each performed at least in 6-replicates.

### Statistical analysis of biological evaluation

Data reported were statistically validated using Student’s t-test comparing mean absorbance of treated and untreated samples. The significance of differences between mean absorbance values was calculated using a two-tailed Student’s t-test. A p value of <0.05 was considered significant.

### Chemical composition analysis of active selected essential oils

EOs were purchased from Farmalabor srl (Assago, Italy) and analyzed to characterize their composition as following.

Chemical analyses of EOs were performed by a Turbomass Clarus 500 GC-MS/GC-FID from Perkin Elmer instruments (Waltham, MA, USA) equipped with a Stabilwax fused-silica capillary column (Restek, Bellefonte, PA, USA) (60 m × 0.25 mm, 0.25 mm film thickness). The operating conditions used were as follows: GC oven temperature was kept at 40 °C for 5 min and programmed to 220 °C at a rate of 6 °C/min, and kept constant at 220 °C for 20 min. Helium was used as carrier gas (1.0 mL/min). Solvent delay 0–2 min and scan time 0.2 s. Mass range was from 30 to 350 m/z using electron-impact at 70 eV mode. 1 μL of each essential oil was diluted in 1 mL of methanol and 1 μL of the solution was injected into the GC injector at the temperature of 280 °C. Relative percentages for quantification of the components were calculated by electronic integration of the GC-FID peak areas. The identification of the constituents was achieved by comparing the obtained mass spectra for each component with those reported in mass spectra Nist and Wiley libraries. Linear retention indices (LRI) of each compound were calculated using a mixture of aliphatic hydrocarbons (C8-C30, Ultrasci) injected directly into GC injector at the same temperature program reported above.

### Determination of EOs minimal inhibitory concentration (MIC)

The MIC was determined as the lowest concentration at which the observable bacterial growth was inhibited. MICs were determined according to the guidelines of Clinical Laboratory Standards Institute (CLSI^33^). Each EO was solubilized by adding DMSO, to generate a mother stock solutionof 1 g/mL. Appropriate dilution (10^6^ cfu/mL) of bacterial culture in exponential phase was used. Antimicrobial activity of each EO was evaluated at a concentration of 1 mg/mL range. Experiments were performed in quadruplicate.

## Unsupervised machine learning clusterization of clinical isolates

The cluster analysis was implemented in the Python (version 3.6) programming language^13^. The *S. aureus* and *P. aeruginosa* datasets were imported in a jupyter-notebook (version 5.7)^34^ and the categorical variables loaded into a Pandas^35^ dataframe were transformed into dummy indicator variables for the subsequent Principal Component Analysis (PCA) using the utilities available in the Pandas (version 0.23) library. The PCA analysis was performed using the scikit-learn library (version 0.20)^36^ to extract the first 20 principal components (PCs, Fig. 1S). The scores and loadings were graphically inspected on plots generated using the matplotlib library (version 3.0)^37^ (Fig. 2S). The PCs were used as features for the k-means clusterization. Silhouette analysis^16^ was performed to evaluate the separation distance between the resulting clusters and choose an optimal value for the number of clusters. Optimal number of clusters was identified by the maximum silhouette scores as graphically reported in Fig. 3S. Through *k*-means, the centroid of each cluster was calculated and the closest datapoint directly indicated the RS (Fig. 4S).

## Supplementary information

Supplementary information.
