# Supplementary material for: Essential oils against bacterial isolates from cystic fibrosis patients by means of antimicrobial and unsupervised machine learning approaches
Source: Sci Rep. 2020 Feb 14;10:2653. doi: 10.1038/s41598-020-59553-8 (PMC7021809; doi:10.1038/s41598-020-59553-8)
Supplement: Supplementary file 1 — Supplementary information. [file 41598_2020_59553_MOESM1_ESM.pdf]

Supplementary Information

Essential oils against bacterial isolates from cystic fibrosis patients by means of antimicrobial and unsupervised machine learning approaches

Rino Ragno, Rosanna Papa, Alexandros Patsilinakos, Gianluca Vrenna, Stefania Garzoli, Vanessa Tuccio, Ersilia Vita Fiscarelli, Laura Selan and Marco Artini

Table 1S. Percentage of susceptibility of *S.aureus* and *P. aeruginosa*

**Classes of antimicrobials**	**Molecules**	**Susceptibility according to EUCAST** [a]					
		S.aureus			***P.aeruginosa***		
		% S	% I	% R	% S	% I	% R
Quinolones	CI	60	5	35	60	-	40
	LE	60	5	35	55	-	45
Sulfonamides	T/S	100	-	-	Nt		
Macrolides	ER	10	10	80	Nt		
Lincosamides	CLI	25	5	70	Nt		
Oxazolidinones	LIN	100	-	-	Nt		
Carbapenems	IP	Nt			55	-	45
	MP	Nt			60	5	35
Penicillins+ β lactamase inhibitors	PTC	Nt			80	-	20
Aminoglycosides	TM	Nt			50	5	45
	AK	Nt			50	5	45
Monobactams	AT	Nt			45	45	10
Cephalosporins	CAZ	Nt			60	-	40
	PM	Nt			60	-	40
Polymyxins	CO	Nt			100	-	-

S, susceptible; I, intermediate; R, resistant; CI, ciprofloxacin; LE, levofloxacin; T/S, trimethoprim/sulfamethoxazole; ER, erythromycin; CLI, clindamycin; LIN, linezolid; IP, imipenem; MP, meropenem; PTC, piperacillin-tazobactam; TM, tobramycin; AK, amikacin; AT, aztreonam; CAZ, ceftazidime; PM, cefepime, CO, colistin. Nt, non-tested.

[a] EUCAST breakpoints (in milligrams per liter) for *S.aureus*: CI-5µg , S ≤ 1 - R > 1; LE-5µg , S ≤ 1 - R > 1;T/S -1.25/23.75 µg , S ≤ 4 - R > 4; ER-15 µg , S ≤ 1 - R > 2; CLI-2 µg , S ≤ 0.25 - R > 0.5; LIN-10 µg , S ≤ 4 - R > 4;

[a] EUCAST breakpoints (in milligrams per liter) for *P.aeruginosa*: CI-5µg , S ≤ 0.5 - R > 0.5; LE-5µg , S ≤ 1 - R > 1; IP-10 µg , S ≤ 4 - R > 8; MP-10 µg , S ≤ 2 - R > 8; PTC –100/10 µg, S ≤ 16 - R > 16; TM –10 µg, S ≤ 4 - R > 4; AK–30 µg, S ≤ 8 - R > 16; AT–30 µg, S ≤ 1 - R > 16; CAZ–30 µg, S ≤ 8 - R > 8; PM–30 µg, S ≤ 8 - R > 8; CO–10 µg, S ≤ 2 - R > 2.

Table 2S. Essential oils names.

EO ID	EO Name	EO ID	EO Name
1	Chamomile Morocco Essential Oil	32	Birch Essential Oil
2	Sage Sclarea Essential Oil	33	Fennel Essential Oil
3	Salvia Officinalis Essential Oil	34	Cedar Fruit Essential Oil
4	Red Thyme Essential Oil	35	Lemon Essential Oil
5	Tea Tree Oil	36	Roman Chamomile Essential Oil
6	Melissa Oiio Essential	37	Savory Essential Oil
7	Pinus Mugo Essential Oil	38	Rosemary Essential Oil
8	Geranium Bourbon Essential Oil	39	Ceylon Cinnamon Peel Essential Oil
9	Oregano Essential Oil	40	Eucaliptus Globulus Essential Oil
10	Ylang Ylang Essential Oil	41	Sweet Orange Essential Oil
11	Coriander Essential Oil	42	Niaouly Essential Oil
12	Lavandula Angustifoglia Essential Oil	43	Artemisia Essential Oil
13	Myrtle Essential Oil	44	Cajeput Essential Oil
14	Garlic Essential Oil	45	Black Pepper Essential Oil
15	Cardamom Essential Oil	46	White Thyme Essential Oil
16	Mandarin Essential Oil	47	Marjoram Essential Oil
17	Hyssop Essential Oil	48	Cloves Essential Oil
18	Grapefruit Essential Oil	49	Cypress Essential Oil
19	Cymbopogon Essential Oil	50	Nutmeg Natural Essential Oil
20	Pinus Sibirica Essential Oil	51	Peppermint Essential Oil
21	Camphor Essential Oil	52	Verbena officinalis Essential Oil
22	Cadè Essential Oil	53	Basil Essential Oil
23	Cedar Leaves Essential Oil	54	Cymbopogon martinii Essential Oil
24	Ginger Essential Oil	55	Laurel Essential Oil
25	Cumin Essential Oil	56	Anise Essential Oil
26	Patchouli Essential Oil	57	Incense Essential Oil
27	Bitter Orange Essential Oil	58	Mentha Suaveolens (Sicily) Essential Oil
28	Eucalyptus Essential Oil	59	Coridotthymus Capitatus (Sicily) Essential Oil
29	Pinus Silvester Essential Oil	60	Thymus Vulgaris (Sicily) Essential Oil
30	Bergamot Essential Oil	61	Origanum Hirtum (Sicily) Essential Oil
31	Juniper Essential Oil		

Table 3S: Chemical composition (%) of Birch EO.

# [1]	Name	RI[2]	RI[lit3]	Area %
**1**	2-cyclopenten-1-one, 3-methyl	1510	1513	0.5
**2**	2-cyclopenten-1-one, 2,3-dimethyl	1528	1535	0.7
**3**	α -cedrene	1590	1599	9.9
**4**	dihydrocurcumene	1610	*	3.5
**5**	isoledene	1655	*	5.7
**6**	α -muurolene	1685	1690	4.4
**7**	δ-cadinene	1758	1758	22.2
**8**	calamenene	1802	1804	15.2
**9**	α-methylnaphtalene	1889	1891	0.9
**10**	guaiacol	1895	1897	3.5
**11**	isolongifolene, 4,5,9,10-dehydro-	1920	*	1.8
**12**	creosol	1960	1956	12.8
**13**	o-creosol	2009	2011	0.9
**14**	phenol	2011	2012	1.1
**15**	p-ethylguaiacol	2027	2032	5.0
**16**	m-cresol	2075	2081	1.4
**17**	phenol, 2,5-dimethyl	2080	2085	1.8
**18**	p-propylguaiacol	2102	2103	2.1
**19**	eugenol	2155	2166	0.9
**20**	cadalene	2199	2200	4.6
**21**	isoeugenol	2270	2268	0.9
	Unidentified compounds			0.2

# indicates the compound identification number; [2] Retention indices measured on polar column; [3] Retention indices from literature; *RI[1lit] not available for polar column. [+]Normal alkane RI;

Table 4S: Chemical composition (%) of Ceylon Cinnamon EO

# [1]	Name	RI[2]	RI[lit3]	Area %
**1**	α –pinene	1021	1021	0.9
**2**	camphene	1060	1065	0.3
**3**	β-pinene	1100	1105	0.3
**4**	α –phellandrene	1161	1160	0.9
**5**	limonene	1200	1198	0.5
**6**	β-phellandrene	1203	1204	1.4
**7**	m-cymene	1260	1258	2.1
**8**	α-copaene	1490	1487	0.4
**9**	β-linalool	1535	1537	5.2
**10**	terpinen-4-ol	1600	1603	0.3
**11**	β-caryophyllene	1617	1619	4.7
**12**	cinnamaldheyde, o-methoxy-	1650	*	1.0
**13**	humulene	1665	1668	0.8
**14**	α-terpineol	1677	1675	0.4
**15**	safrole	1870	1874	0.3
**16**	cinnamaldheyde	2037	2049	49.4
**17**	eugenol	2170	2175	21.2
**18**	eugenol acetate	2270	2277[+]	0.9
**19**	benzyl benzoate	2648	2652	8.6
	Unidentified compounds			0.4

# indicates the compound identification number; [2] Retention indices measured on polar column; [3] Retention indices from literature; *RI[Ilit] not available for polar column. [+]Normal alkane RI.

Table 5S: Chemical composition (%) of Cadè EO

# [1]	Name	RI[2]	RI[lit3]	Area %
**1**	2-cyclopenten-1-one, 3-methyl	1510	1513	0.7
**2**	α-cedrene	1590	1599	7.9
**3**	aromadendrene	1609	1610	1.1
**4**	dihydrocurcumene	1690	1696	3.5
**5**	α-selinene	1751	1750	2.2
**6**	α-muurolene	1755	*	4.0
**7**	δ-cadinene	1758	1758	27.7
**8**	calamenene	1802	1832	14.8
**9**	phenol, 2-methoxy-	1838	1846	4.7
**10**	isolongifolene, 4,5,9,10-dehydro-	1920	*	1.7
**11**	creosol	1960	1956	12.6
**12**	o-creosol	2009	2011	1.0
**13**	phenol	2011	2012	1.0
**14**	p-ethylguaiacol	2027	2032	5.4
**15**	m-cresol	2075	2081	1.5
**16**	phenol, 3-methyl-	2095	2099	1.1
**17**	p-propylguaiacol	2102	2103	1.9
**18**	eugenol	2155	2175	0.4
**19**	gleenol	2175	*	1.1
**20**	cadalene	2199	2200	4.3
**21**	isoeugenol	2270	2268	1.2
	Unidentified compounds			0.2

# indicates the compound identification number; [2] Retention indices measured on polar column; [3] Retention indices from literature; *RI[Ilit] not available for polar column. [+]Normal alkane RI;

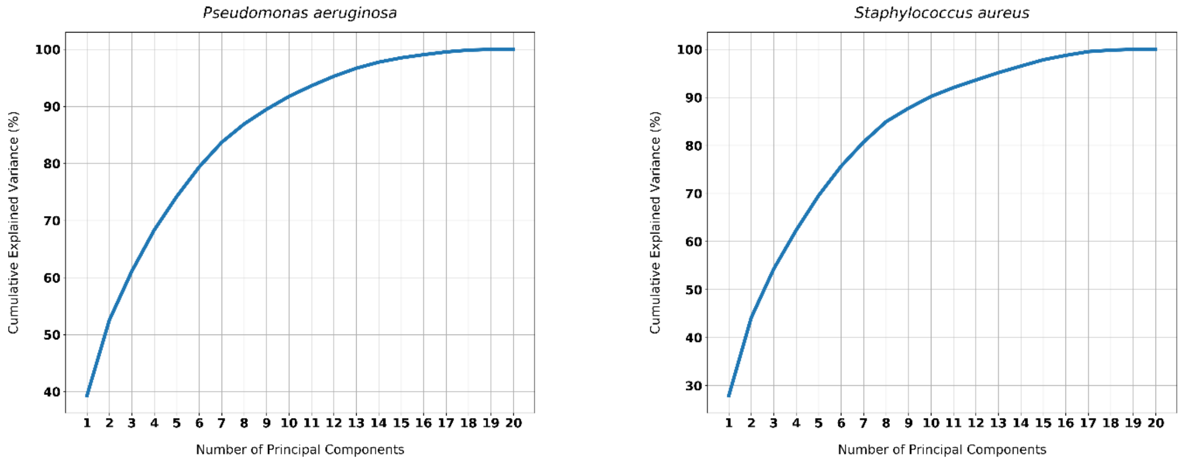

Figure 1S. Explained cumulative variance versus the number of extracted PCs for the PA (left panel) and SA (right panel) datasets

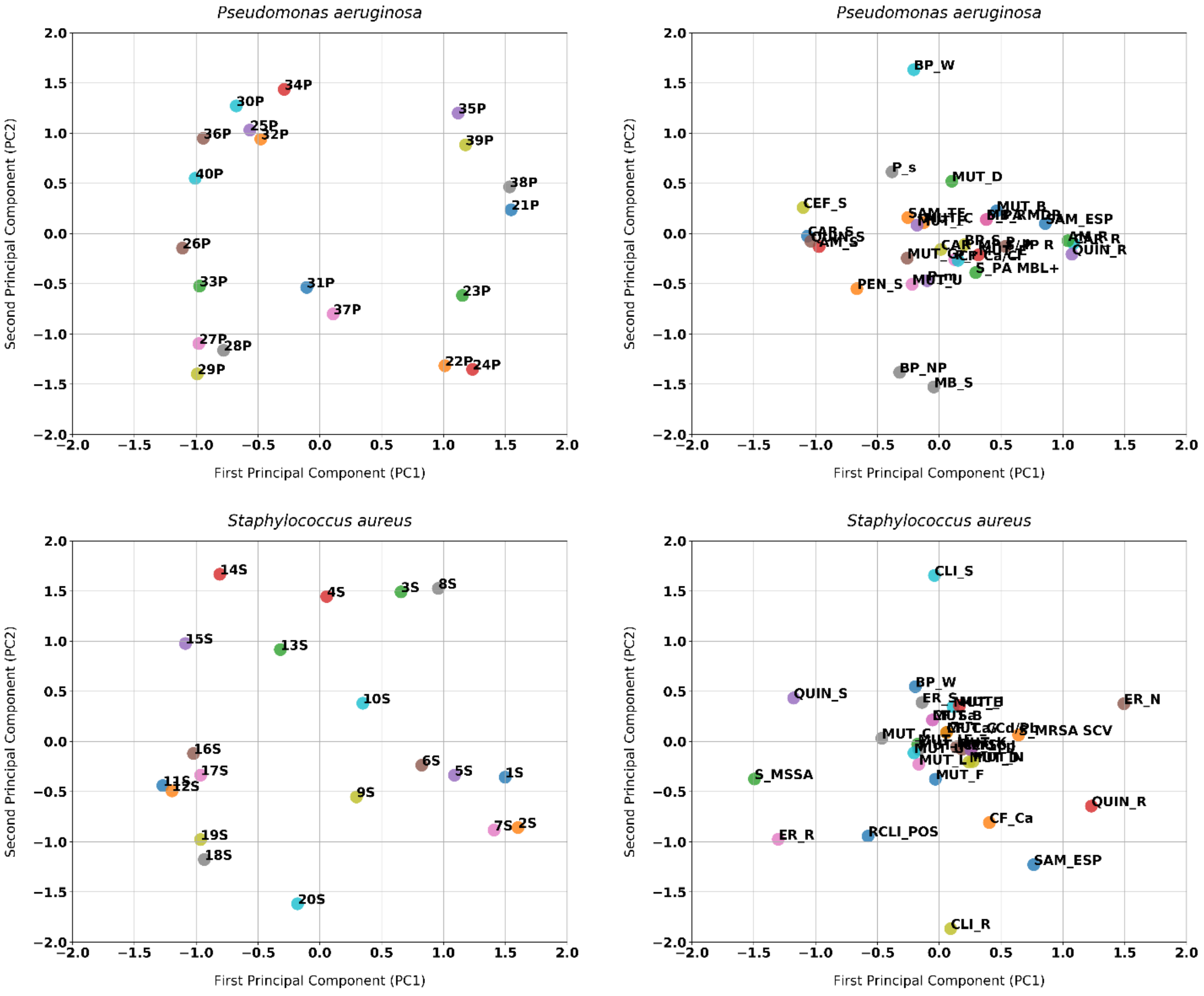

Figure 2S. Scores (left panels) and loadings (right panels) plots for the PA (Top Panels) and SA (bottom panels) datasets

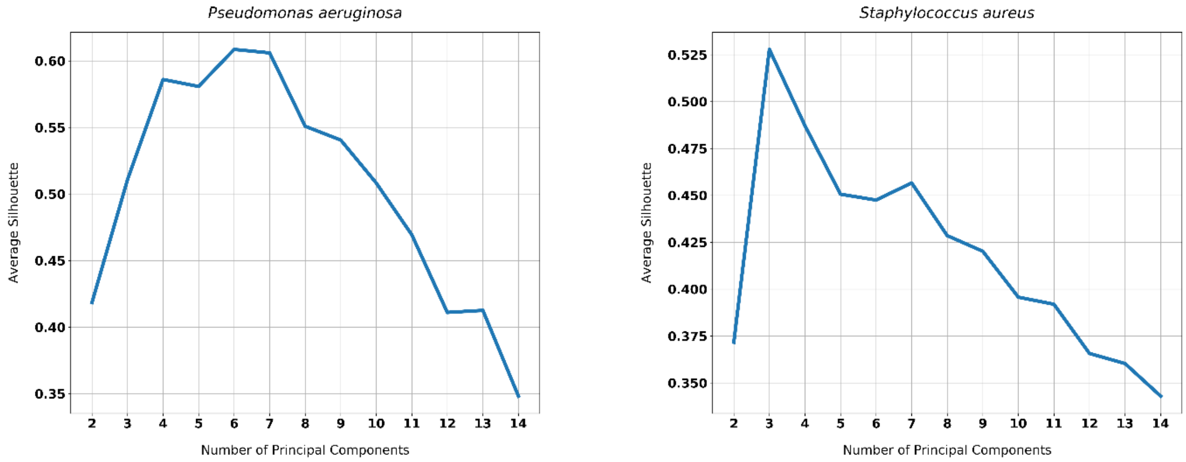

Figure 3S. Average silhouette scores versus the number of clusters. The maximum indicate the optimal number of clusters.

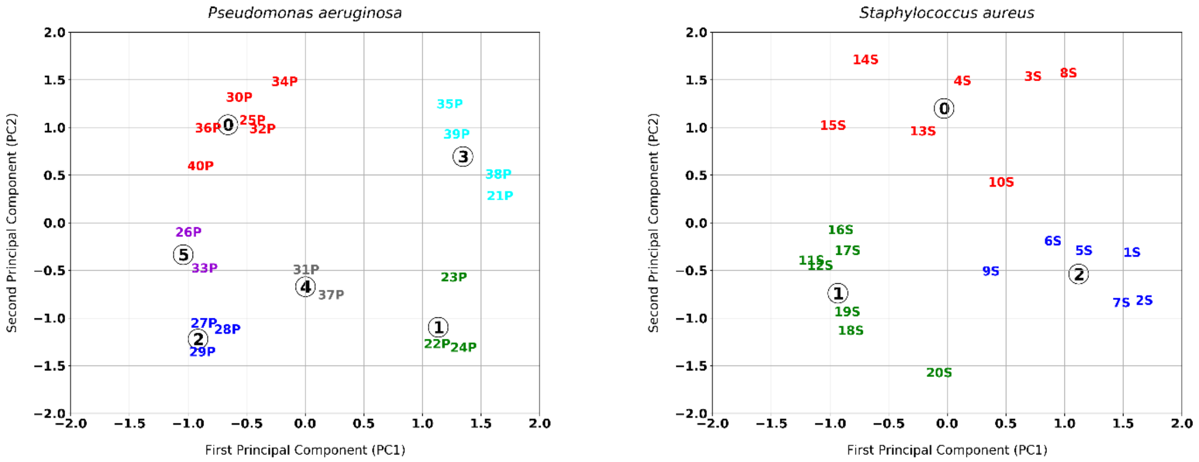

Figure 4S. Score plots indicating the clustered PA (left panel) and SA (right panel) datasets. The k-means centroids of each cluster are also reported.
